# Psychometric Properties of the Socio-Emotional Competence Assessment Scale (ECSE) in the Cuban Context

**DOI:** 10.11621/pir.2025.0203

**Published:** 2025-05-01

**Authors:** Marena de la Caridad Hernández-Lugo, Annia Esther Vizcaíno Escobar, Evelyn Fernández Castillo, Diego D. Díaz-Guerra, Mario Pena Garrido

**Affiliations:** a Central University “Marta Abreu” of Las Villas, Villa Clara, Cuba; b National University of Distance Education, Spain

**Keywords:** socio-emotional competencies, psychometric properties, validation, Cuban population, university students

## Abstract

**Background:**

Socio-emotional competencies are important for mental health and successful human interaction. In the university context, strengthening socio-emotional competencies is essential for fostering a collaborative learning environment, where students can manage academic stress and build meaningful connections with their peers.

**Objective:**

To test the psychometric properties of the Socio-Emotional Competence Assessment Scale in the Cuban population.

**Design:**

Two cross-sectional studies were conducted. Study 1 (N = 640) assessed the psychometric properties of the Socio-Emotional Competence Assessment Scale within the Cuban population. In Study 2 (N = 913), the nomological validity of the scale was evaluated.

**Results:**

In Study 1, the scale demonstrated high internal consistency and the expected three-factor structure. We had to eliminate one item, as it was negatively correlated with the rest of the scale items; the result was the Cuban ECSE. In Study 2, we tested the nomological validity of the scale: we found that socio-emotional competencies had medium-sized positive correlations with subjective well-being and perceived academic self-efficacy. Negative associations were found between socio-emotional competencies and academic stress. Additionally, socio-emotional competencies were positively and statistically significantly correlated with the dimension of coping strategies in response to stressful stimuli or situations.

**Conclusion:**

The use of the ECSE is recommended in the Cuban context.

## Introduction

Socio-emotional competencies play a fundamental role in academic development, social interaction, emotional well-being, and the overall success of individuals (Guo et al., 2021; Huang & Zeng, 2023). These competencies not only influence how students relate to others, but also their ability to face challenges, regulate their emotions, resolve conflicts, and adapt to different environments (Malinauskas & Malinauskiene, 2021; Santamaría-Villar et al., 2021).

In the university context, where young people face new responsibilities, academic demands, and life experiences, socio-emotional competencies become even more relevant (Gamboa et al., 2023; Merlin & Soubramanian, 2024; Portela-Pino et al., 2021). University students need not only solid academic knowledge, but also skills to communicate effectively, work in teams, manage stress, resolve conflicts, and maintain a positive attitude in the face of challenges (Chen, 2022; Talwar et al., 2022).

Considering the relationship between socio-emotional competencies and other significant variables in the university context, previous research has shown that students with a higher level of socio-emotional competencies have better academic self-efficacy (Huang & Zeng, 2023; Portela-Pino et al., 2021; Shafait et al., 2021). Furthermore, this construct has been consistently associated with lower levels of academic stress among students (Koppenborg et al., 2022; Wunsch et al., 2021). Studies such as those by Arteaga-Cedeño et al. (2022), Morales-Rodríguez et al. (2020), and Portela-Pino et al. (2021) found that socio-emotional competencies are positively related to the psychological and subjective well-being of students.

Several studies have demonstrated that socio-emotional competencies are positively related to individuals’ psychological well-being (Anderson et al., 2023; Barrera et al., 2019; Danner et al., 2021; Morales-Rodríguez et al., 2020). Individuals with high socio-emotional competencies tend to experience greater life satisfaction, lower levels of anxiety and depression, and greater resilience in the face of emotional and academic challenges (Abrahams et al., 2019; Costa et al., 2020).

Socio-emotional competencies have been consistently associated with lower levels of academic stress among students (Ullah et al., 2023; Vestad & Tharaldsen, 2021; Yu et al., 2021). Those who are able to regulate their emotions, manage stress, and establish positive relationships tend to experience less anxiety related to academic demands, which in turn can favor better academic performance (Vestad et al., 2021; Wang et al., 2019).

Academic self-efficacy, which refers to the belief in one’s ability to successfully tackle academic tasks and achieve educational goals, is also influenced by socio-emotional competencies (Huang & Zeng, 2023; Portela-Pino et al., 2021; Supervía & Robres, 2021). Individuals with high socio-emotional competencies often have greater academic self-efficacy, allowing them to face academic challenges with confidence and persistence. This contributes to better academic performance and greater satisfaction with their achievements (Cheng, 2020; Kim & Shin, 2021; Primi et al., 2021).

Given the importance of socio-emotional competencies in the university context, it is crucial to have validated and reliable tools to assess these competencies in university students (Cavioni et al., 2023; Cronin et al., 2019). The Socio-Emotional Competence Assessment Scale (ECSE) is presented as an instrument specifically designed to measure these skills in the young population (Repetto Talavera, 2009). Despite the excellent psychometric properties of the scale and its added value for assessing socio-emotional competencies in young populations, there are currently no adaptations available for other Latin American contexts. Furthermore, the Cuban population lacks a validated instrument to evaluate these competencies in young individuals.

Adaptation of the ECSE scale to the Cuban context becomes essential not only because there are currently no locally validated instruments to assess socio-emotional competencies in youth, but also due to Cuba’s unique cultural and educational particularities. On the one hand, factors such as the emphasis on collective values, the free education system, and historical resilience (Nartova-Bochaver et al., 2022) may influence the manifestation of certain competencies, which requires verification of the items’ conceptual equivalence. On the other hand, without rigorous linguistic and psychometric adaptation, the instrument could lose ecological validity by failing to capture these nuances (Uysal-Bozkir et al., 2013). Furthermore, having an adapted version would establish the foundation for future validations in the Latin American context and enable the generation of local normative data useful for future research and the design of educational policies tailored to the country’s actual needs.

Therefore, the objective of this research is to evaluate the psychometric properties of the Socio-Emotional Competence Assessment Scale in the Cuban university population. To achieve this objective, two studies were conducted to assess the psychometric properties of the scale. In Study 1, the factorial structure of the instrument was explored through a construct validity analysis. Study 2 aimed to evaluate the nomological validity of the scale by examining the association of socio-emotional competencies with (a) perceived academic self-efficacy, (b) psychological and subjective well-being, and (c) academic stress. The databases for both studies are publicly available in the Mendeley Data repository [https://doi.org/10.17632/c6c2tngbhd.1].

## Study 1 — Construct Validity

### Methods1

#### Participants

A cross-sectional study was conducted using an online survey through the Google Forms platform from January to December 2024. To ensure probabilistic sampling, simple random sampling was employed. A random sample of Cuban citizens over the age of 18 who were pursuing university studies within the country was selected, ensuring that all members of the eligible population had an equal chance of being chosen. The survey was disseminated through WhatsApp groups, Facebook^*^, email lists, and websites. No incentives were offered for participation. A total of 640 students participated (345 women and 295 men), with a mean age of 20.84 (*SD* = ±1.96). The study protocol was approved by the Ethics Committee of the Department of Psychology at the Central University “Marta Abreu” of Las Villas.

#### Procedure

##### Questionnaires

*Demographic Variables:* The demographic variables explored included age, gender, and the institution where the university studies were being pursued.

*Socio-Emotional Competence Assessment Scale (ECSA):* This scale was designed and validated by Repetto Talavera (2009) for Spanish-speaking participants. It consists of 38 Likert-type items ranging from 1 (“not identified at all”) to 5 (“fully identified”) that assess socio-emotional competencies across seven dimensions: Self-Awareness (items 3, 14, 28, 34, 32), Emotional Self-Regulation (items 1, 8, 16, 27, 35), Interpersonal Regulation (items 6, 23, 30, 19, 9, 11), Empathy (items 15, 18, 25, 24, 20, 26), Motivation (items 21, 33, 36, 22, 5), Conflict Resolution (items 2, 10, 4, 7, 31), and Teamwork (items 13, 17, 37, 28, 29). Items 9, 16, 27, and 31 are to be evaluated in reverse. The linguistic adaptation to the Cuban context was carried out by Chou-Ramírez et al. (2023) based on expert criteria.

##### Data Analysis Procedure

Data were processed using JASP statistical software version 0.18.3.0. Descriptive analyses were employed to understand the characteristics of the participants, and confirmatory factor analysis (CFA) was used to evaluate the factorial structure of the ECSE. Pearson correlation analyses were conducted to assess the relationships among the socio-emotional competencies that comprise the instrument, considering that correlation indices of *r* ≤ .10 were regarded as small, *r* = .20 as moderate, and *r* ≥ .30 as large (Funder & Ozer, 2019).

Acceptable values for the Comparative Fit Index (CFI) and the Tucker-Lewis Index (TLI) were assumed to be between .90 and .95, and those for the Standardized Root Mean Square Residual (SRMR) were considered optimal at < .08 and acceptable at ≤ .10 (Hu & Bentler, 1999). For the Root Mean Square Error of Approximation (RMSEA), values below .10 were considered acceptable (Xia & Yang, 2019). Cron-bach’s alphas and McDonald’s omegas were used to evaluate reliability, with values equal to or greater than .70 indicating good internal consistency (Dunn et al., 2013; Kelley, 2018; Shrestha, 2021).

To assess normality, the Kolmogorov-Smirnov test was employed. The obtained *p*-values were less than .05, indicating that the items of the ECSE do not follow a normal distribution. The analysis of skewness and kurtosis revealed values exceeding ±1, providing conclusive evidence that the data do not follow a normal distribution (Mishra et al., 2019). Given the lack of normality in the sample, the Weighted Least Squares Mean and Variance adjusted (WLSMV) estimator was used for the CFA (Xia & Yang, 2019).

### Results

A total of 640 Cuban university students were evaluated, comprising 345 women and 295 men, with a mean age of 20.84 (Range = 18–33, *SD* = ±1.96). The expected seven-factor solution demonstrated indices that were not entirely satisfactory according to the expected results, χ^2^(644) = 2805.350, *p* < .001; RMSEA = .072, 90% CI [.070, .075], *p* < .001; CFI = .952; TLI = .947; SRMR = .09. Upon examining the modification indices, one item was identified as having low fit indicators to the model (ECSE-16 “Sometimes I can’t help but get nervous”). Additionally, this item showed a negative factor loading (–.08) and a low item-test correlation (r = .06), leading to the decision to exclude it from the factor solution.

The remaining factor solution, consisting of 37 items and 7 dimensions, showed satisfactory fit values, χ^2^(608) = 2274.249, *p* < .001; RMSEA = .065, 90% CI [.063, .068], *p* < .001; CFI= .962; TLI = .959; SRMR = .09. The new item-total correlation was acceptable, and all factor loadings were significant (*p* < .001). *Table 1* presents the mean values, standard deviations, corrected item-total correlations, and factor loadings for each item. The items exhibited approval rates above the expected average and a considerable level of variance.

**Table 1:** Construct Validity Analysis

Items	*M*	*SD*	*r*	*L*
**F1 – Self-Awareness**				
ECSE-3 Cuando estoy enfadado, normalmente se cuál es la causa de mi enfado / *When I am angry, I usually know the cause of my anger.*	4.58	.70	.42	.72
ECSE-14 Normalmente puedo identificar la razón de lo que siento / *Ican usually identify the reason for what I feel.*	4.37	.77	.47	.79
ECSE-28 La mayoría de las veces puedo explicar por qué cambian mis emociones / *Most of the time, I can explain why my emotions change.*	4.13	.90	.45	.74
ECSE-34 Cuando me sucede algo, sea bueno o malo, sé cómo me siento / *When something happens to me, whether good or bad, I know how I feel.*	4.53	.67	.47	.83
ECSE-32 Entiendo bien mis emociones / *I have a good understanding of my emotions.*	4.30	.83	.52	.89
**F2 - Emotional Self-Regulation**				
ECSE-1 Cuando estoy enfadado/a puedo tranquilizarme con facilidad / *When I am angry, I can calm myself down easily.*	4.04	.88	.35	.71
ECSE-8 Soy capaz de mantener un buen estado de ánimo a pesar de los problemas que me pasan / *I am able to maintain a good mood despite the problems I face.*	4.23	.90	.39	.72
ECSE-27 Cuando tengo problemas siento que no puedo controlar mis emociones / *When I have problems, I feel like I cannot control my emotions.*	2.16	.96	.12	.42
ECSE-35 Yo sé cómo mantener la calma durante una discusión / *I know how to stay calm during a discussion.*	4.10	.89	.37	.78
**F3 - Interpersonal Regulation**			
ECSE-6 Cuando mis amigos están de mal humor, se cómo calmarlos / *When my friends are in a bad mood, I know how to calm them down.*	4.19	.81	.50	.73
ECSE-23 Si un amigo está nervioso soy capaz de tranquilizarlo / *If a friend is nervous, I can help them relax.*	4.15	.75	.54	.79
ECSE-30 Cuando un amigo se siente bien, yo sé cómo hacer para que continúe con este ánimo / *When a friend is feeling good, I know how to help them maintain that mood.*	4.46	.75	.57	.85
ECSE-19 Cuando alguien está triste sé cómo ayudarle para que se sienta mejor / *When someone is sad, I know how to help them feel better.*	4.19	.79	.54	.84
ECSE-9 Cuando un amigo está deprimido no sé qué hacer con él / *When a friend is depressed, I don’t know what to do with them.*	2.47	.94	.20	.36
ECSE-11 Sé cómo ayudar a las personas que se sienten mal / *I know how to help people who are feeling bad.*	4.25	.79	.46	.76
**F4 – Empathy**				
ECSE-15 Trato de comprender a mis amigos poniéndome en su lugar / *I try to understand my friends by putting myself in their shoes.*	4.59	.68	.58	.81
ECSE-18 Estoy seguro de que los demás saben que los comprendo / *I am sure that others know I understand them.*	4.05	.90	.52	.69
ECSE-25 Trato de buscar las palabras adecuadas para que el otro se sienta comprendido / *I try to find the right words so that the other person feels understood.*	4.57	.69	.56	.77
ECSE-24 Miro a los ojos de la persona que me está hablando para comprenderla mejor / *I look into the eyes of the person who is speaking to me to understand them better.*	4.49	.79	.38	.54
ECSE-20 Al escuchar el problema de un compañero me imagino como me sentiría yo / *When listening to a classmate’s problem, I imagine how I would feel.*	4.57	.73	.45	.68
ECSE-26 Creo que soy una persona sensible hacia los otros / *I think I am a person who is sensitive toward others..*	4.24	.93	.42	.58
**F5 – Motivation**				
ECSE-21 En mis tareas académicas tengo la curiosidad de saber cada vez más / *In my academic tasks, I am curious to learn more and more.*	4.15	.88	.46	.74
ECSE-33 Me encanta aprender cosas nuevas / *I love learning new things.*	4.71	.57	.48	.80
ECSE-36 Me gusta hacer bien las cosas porque disfruto haciéndolas y no porque me felicitan / *I like to do things well because I enjoy doing them, not just because I receive praise.*	4.59	.68	.49	.75
ECSE-22 Es fácil para mi tener interés para estudiar o hacer cosas / *It is easy for me to have an interest in studying or doing things.*	3.96	.95	.44	.73
ECSE-12 Me gusta ser responsable con mis tareas / *I like to be responsible for my tasks.*	4.36	.89	.41	.73
ECSE-5 Cuando tengo dificultades en mi estudio trato de hacer todo para solucionarlas / *When I have difficulties in my studies, I try to do everything to solve them.*	4.34	.83	.43	.74
**F6 - Conflict Resolution**				
ECSE-2 Cuando un compañero se enfrenta a un problema, le ayudo a pensar en soluciones / *When a classmate faces a problem, I help them think of solutions.*	4.74	.55	.51	.79
ECSE-10 Cuando tengo una discusión trato de dialogar y tener en cuenta el punto de vista de los demás / *When I have a disagreement, I try to have a dialogue and consider others’ viewpoints.*	4.41	.78	.51	.65
ECSE-4 Cuando tengo desacuerdos con alguien, trato de buscar soluciones que nos favorezcan a todos / *When I have disagreements with someone, I try to find solutions that benefit us all.*	4.43	.76	.52	.67
ECSE-7 Cuando entre mis compañeros se produce un conflicto trato de conocer qué piensan cada uno de ellos / *When a conflict arises among my peers, I try to understand what each of them thinks.*	4.39	.90	.45	.64
ECSE-31 Para mí es difícil ayudar a solucionar un conflicto entre dos personas / *It is difficult for me to help resolve a conflict between two people.*	2.25	.91	.13	.19
**F7 – Teamwork**				
ECSE-13 Me gusta trabajar en equipo / *I enjoy working in a team.*	4.06	.99	.39	.56
ECSE-17 Logro motivar a mis compañeros para que se integren en las tareas del equipo / *I am able to motivate my teammates to engage in team tasks.*	3.91	.96	.52	.72
ECSE-37 Cuando trabajo con mi grupo me comprometo mucho con lo que tenemos que hacer / *When I work with my group, I commit fully to what we need to do.*	4.53	.68	.49	.75
ECSE-38 Cuando trabajamos en equipo los buenos resultados son de todos / *When we work as a team, the good results belong to everyone.*	4.64	.70	.30	.53
ECSE-29 Trabajo con mis compañeros de forma cooperativa, compartiendo ideas, información y soluciones */ I work cooperatively with my peers, sharing ideas, information, and solutions.*	4.40	.81	.54	.77

*Note. M = mean, SD = standard deviation, r = item-total correlation, L = factor loadings.*

Additionally, statistically significant correlations of moderate to large magnitudes were found among the factors of the scale (see *Table 2*). Overall, it is observed that all competencies are significantly correlated with each other, with correlation values ranging from .151 to .621, all at a significance level of *p* < .01. This suggests that as one competency strengthens, the others also tend to improve.

**Table 2:** Descriptive and Correlation Matrix of Socio-Emotional Competencies

	*M* (SD)			F 1	F 2	F 3	F 4	F 5	F 6	F 7
	General	Female	Male							
Factor 1: Self-Awareness	1.22 (.41)	1.19 (.39)	1.25 (.43)	–						
Factor 2: Emotional Self-Regulation	1.42 (.49)	1.33 (.47)	1.53 (.50)	.441**	–					
Factor 3: Interpersonal Regulation	1.37 (.60)	1.37 (.61)	1.38 (.60)	.318**	.185**	–				
Factor 4: Empathy	1.18 (.43)	1.22 (.46)	1.15 (.39)	.359**	.151**	.592**	–			
Factor 5: Motivation	1.31 (.50)	1.35 (.51)	1.28 (.49)	.379**	.270**	.271**	.391**	–		
Factor Resolution 6: Conflict	1.41 (.63)	1.44 (.64)	1.37 (.61)	.339**	.259**	.526**	.621**	.397**	–	
Factor 7: Teamwork	1.18 (.43)	1.19 (.44)	1.17 (.42)	.291**	.213**	.389**	.547**	.511**	.477**	–
Age				–.02	.05	.03	–.07	–.08	.01	–.03
Gender				.08	.20**	.00	–.08	–.07	–.05	–.03

Note. M = mean, SD = standard deviation, ** = p < .01.

Furthermore, the seven-factor model was compared with a unidimensional model in which all items loaded onto a single latent factor of socio-emotional competencies, as well as a hierarchical model where the items loaded onto their corresponding dimensions, and these dimensions loaded onto a global factor of socio-emotional competencies. The hierarchical model showed good fit, χ^2^(622) = 3154.978, *p* < .001; RMSEA = .080, 90% CI [.077, .083], *p* < .001; CFI = .943; TLI = .938; SRMR = .09, although it was lower than the original seven-factor model without a second order. In contrast, the unidimensional model did not show good fit, χ^2^(629) = 7048.907, *p* < .001; RMSEA = .126, 90% CI [.124, .129], *p* < .001; CFI = .854; TLI = .846; SRMR = .09.

The reliability estimates for the overall scale showed high values (α = .90, ω = .90). Regarding the factor analysis, medium values predominated in self-awareness (α = .84, ω = .83), interpersonal regulation (α = .81, ω = .81), empathy (α = .76, ω = .76), motivation (α = .82, ω = .80), and teamwork (α = .73, ω = .72). There were medium-low values for emotional self-regulation (α = .69, ω = .69) and conflict resolution (α = .60, ω = .58).

Furthermore, normative scores for the scale were calculated for the Cuban university population (see *Table 3*). These scores allow for contextualization and a better understanding of individual results by comparing them with the average performance of Cuban university students.

**Table 3:** Cut-off Scores for the Assessment of Socio-Emotional Competencies

Dimensions	Scores								
	Limited			Moderate			Excellent		
	General	Female	Male	General	Female	Male	General	Female	Male
Factor Awareness 1: Self-	≤ 20	≤20	≤20	21–25	21–23	21–24	26 ≥	≥24	≥25
Factor Self-Regulation 2: Emotional	≤ 13	≤12	≤14	14–16	13–15	15–16	17 ≥	≥16	≥17
Factor 3: Inter-personal Regulation	≤ 22	≤22	≤21	23–26	23–26	22–25	27 ≥	≥27	≥26
Factor 4: Empathy	≤ 25	≤26	≤24	26–29	27–28	25–27	30 ≥	≥29	≥28
Factor 5: Motivation	≤ 24	≤25	≤24	25–29	26–28	25–28	30 ≥	≥29	≥29
Factor Resolution 6: Conflict	≤ 19	≤19	≤18	20–22	20–21	19–21	23 ≥	≥22	≥22
Factor 7: Teamwork	≤ 20	≤20	≤20	21–24	21–23	21–23	25 ≥	≥24	≥24

## Study 2 — Nomological Validity

### Methods

#### Participants

A cross-sectional study was conducted using an online survey through the Google Forms platform from September to November 2024. To ensure probabilistic sampling, simple random sampling was employed, allowing all students from the Central University “Marta Abreu” of Las Villas in the Regular Day Course to have an equal chance of being selected for participation. The survey was disseminated through WhatsApp groups, Facebook, email lists, and websites. No incentives were offered for participation. A total of 913 students participated (481 women and 432 men), with a mean age of 19.21 (*SD* = ±1.45). The study protocol was approved by the Ethics Committee of the Department of Psychology at the Central University “Marta Abreu” of Las Villas.

#### Procedure

##### Questionnaires

*Specific Academic Situations Perceived Self-Efficacy Scale:* Designed by Palenzuela (1983) and validated by Baute Abreu and Vizcaíno Escobar (2023) in the Cuban university population, this scale aims to assess students’ academic self-efficacy through a 10-item questionnaire (e.g., “I consider myself sufficiently capable to successfully face any academic task”), using a 4-point Likert scale ranging from “never” to “always”. In the studied sample, excellent reliability estimates were obtained (α = .90; ω = .90).

*Reduced Subjective Well-Being Scale:* This scale was validated in the Cuban population by Rodríguez-Martín, B and Molerio-Pérez (2012) and consists of 10 items that evaluate the level of subjective well-being perceived by the individual (e.g., “I face my tasks with good spirits”). It uses a Likert scale from 1 (“never or almost never”) to 5 (“always”). In the studied sample, excellent reliability estimates were obtained (α = .83; ω = .83).

*Cognitive-Behavioral Systemic Inventory for the Study of Academic Stress* (SISCO): This instrument represents the second version of the SISCO academic stress inventory, created and validated by Barraza-Macías (2018) based on the original 37-item technique. The scale has a first dimension (SISCO general mean) that evaluates the level of perceived academic stress intensity on a scale from 1 (“little”) to 5 (“a lot”); this data can be used by the researcher as a single-item variable to measure stress intensity or, alternatively, removed. The second dimension (Dimension of Stressors; *e.g*., “The overload of school tasks and assignments I have to complete every day”) assesses the frequency with which environmental demands are perceived as stressors through seven Likert-type items ranging from 0 (“never”) to 5 (“always”). The third dimension (Dimension of Symptoms or Reactions to Stressors, e.g., “anxiety, distress or despair”) evaluates the frequency of symptoms or reactions to a stressor through the same seven items using the previous Likert scale. Finally, the fourth dimension (Dimension of Coping Strategies, e.g., “focusing on solving the situation that worries me”) consists of seven Likert items with the same categorical values aimed at identifying the frequency of coping strategy usage. This scale does not have validation in the Cuban population; therefore, reliability estimates were conducted for the studied sample, yielding acceptable reliability values (α = .84; ω = .85).

#### Data Analysis Procedure

Data were processed using JASP statistical software version 0.18.3.0. Descriptive analyses were employed to understand the characteristics of the participants, and Pearson correlation analyses were conducted to evaluate the relationships between socio-emotional competencies and other variables to verify the nomological validity of the scale, considering that correlation indices of *r* ≤ .10 were regarded as small, *r* = .20 as moderate, and *r* ≥ .30 as large (Funder & Ozer, 2019).

### Results

The sample consisted of 913 students, of whom 52.7% were women and 47.3% were men with an average age of 19.21 (*R* = 18–27, *SD* = ±1.45). There was representation from each of the 12 faculties at the Central University “Marta Abreu” of Las Villas. Of the total participants, 33.8% were in their first year of studies, 29.9% in their second year, 19.5% in their third year, 14.7% in their fourth year, and 2.1% in their fifth year of university.

*Table 4* presents the correlations for the analysis of nomological validity between socio-emotional competencies in the university context and the variables that have theoretically been shown to relate to the studied construct. Direct and significant correlations were observed between all socio-emotional competencies and perceived academic self-efficacy, with the highest correlation index obtained for the motivation dimension. The other dimensions showed moderate correlation values. These data suggest that as socio-emotional competencies increase among university students, perceived academic self-efficacy is also likely to increase.

Direct and significant correlations were also observed between all evaluated socio-emotional competencies and the participants’ subjective well-being. The dimensions of motivation and teamwork exhibited the highest correlation indices, followed closely by the dimension of interpersonal regulation. The remaining dimensions showed moderate to high correlation indices, indicating that as performance in socio-emotional competencies improves, subjective well-being also increases (see *Table 4*).

**Table 4:** Nomological Validity Analysis of the Socio-Emotional Competence Assessment Scale

Measures	Factor 1: Self- Awareness	Factor 2: Emotional Self-Regulation	Factor 3: Inter-personal Regulation	Factor 4: Empathy	Factor 5: Motivation	Factor 6: Conflict Resolution	Factor 7: Teamwork
Perceived Self-Efficacy Academic	.29***	.21***	.29***	.19***	.37***	.22***	.23***
Subjective Well-Being	.26***	.27***	.30***	.24***	.31***	.22***	.31***
General SISCO Mean	–.03	–.16***	.05	.15***	.03	.03	.10**
Dimension ors -SISCO of Stress-	–.14***	–.17***	–.06	.01	–.12***	–.08*	.05
Dimension of Symptoms or Reactions to Stressors -SISCO	–.09**	–.34***	–.03	.05	–.09**	–.04	–.05
Dimension Strategies -SISCO of Coping	.20***	.22***	.19***	.26***	.29***	.20***	.22***

Notes. * p < .05, ** p < .01, *** p < .001.

Regarding the analysis of academic stress and socio-emotional competencies, inverse and significant correlations were found between mean stress scores and emotional self-regulation. Direct and significant correlations were also observed between the general mean of stress and interpersonal regulation and teamwork. This suggests that as students demonstrate better competencies in interpersonal relationships and teamwork, they may also experience higher levels of academic stress, which can be inferred to be due to external social factors (see *Table 4*).

In terms of the presence of stressors, inverse and significant correlations were observed with self-awareness, emotional self-regulation, motivation, and conflict resolution. These socio-emotional competencies were found to be the most negatively influenced by stressors that may arise in the university context (see *Table 4*).

Additionally, particularly in the dimension of symptoms or reactions to stressors, inverse and significant correlations were observed with the socio-emotional competencies of self-awareness, emotional self-regulation, and motivation. These results suggest that the biopsychosocial symptoms that emerge in response to stressors in the university setting negatively impact these socio-emotional competencies (see *Table 4*).

Finally, all evaluated socio-emotional competencies correlated positively and statistically significantly (*p* < .001) with the dimension of coping strategies in response to stressors or stressful situations. In particular, motivation showed the highest correlation index, while the others exhibited moderate to high indices. This allows us to infer that as coping strategies become more adaptive, there will be better performance in the evaluated socio-emotional competencies (see *Table 4*).

## Discussion

This research confirmed the factorial structure of the scale composed of seven factors that assess socio-emotional competencies in young populations. However, to date, no confirmatory factor analyses of the scale have been reported for Cuban university students, making this research foundational for the study and replication of the psychometric properties of the Socio-Emotional Competence Assessment Scale.

Despite this, the present results align with those obtained in the validation of the original scale, which was conducted through an exploratory factor analysis using principal components with Varimax rotation, confirming the presence of seven factors that explained 48.14% of the variance (Repetto Talavera, 2009). The items of the scale showed approval rates above the expected average in both validations, with mean scores ranging from 2.16 to 4.74. Additionally, a considerable level of variance was observed in the responses, with standard deviations ranging from .19 to .96.

However, in the current research, it was necessary to eliminate one item (16, “Sometimes I can’t help but get nervous”) from the 38 items obtained in the original validation due to its negative factor loading and low item-test correlation. This eliminated item has also shown problematic indicators in other studies exploring the psychometric properties of the scale (Chou Ramírez, 2023).

The correlation matrix between the subscales of the ECSE in the original validation showed significant correlation indices for all dimensions of the instrument, except for the relationship between empathy and emotional self-regulation (Repetto Talavera, 2009). These results are similar to those obtained in the present research, as significant correlation indices were also found among all subscales, including those that did not show significance in the original validation. In both cases, the correlations had moderate to high magnitudes.

Regarding the reliability of the scale, high internal consistency values were observed for the overall scale, with acceptable values in most of the evaluated dimensions. These findings are consistent with the study conducted by San Martin and Gromiria (2021), where the reliability of the instrument was high, with a Cronbach’s alpha of .895. In the research by López-Barajas and Reina-Estévez (2012), the reliability by dimensions was as follows: Emotional Self-Awareness (α = .787), Emotional Self-Regulation (α = .642), Interpersonal Emotional Regulation (α = .724), Empathy (α = .724), Motivation (α = .776), Teamwork (α = .748), and Conflict Resolution (α = .645), with the total reliability of the scale equal to .895.

The findings of this research reveal significant correlations between socio-emotional competencies and perceived academic self-efficacy. It is noteworthy that direct correlations were observed between all socio-emotional competencies and academic self-efficacy, with the motivation dimension showing the highest correlation index. Similarly, in the study conducted by Moradi and Chemelnezhad (2021), academic self-efficacy positively and significantly predicted emotional-social competence, indicating that as students’ academic self-efficacy increases, so does their socio-emotional competence. Likewise, the results of the study by Munir et al. (2023) determined a significant association between the variables of socio-emotional competence and self-efficacy.

The results obtained by Çıkrıkçı and Odacı (2016) regarding a positive and significant association between self-awareness and self-efficacy in adolescents are consistent with those found in this study, where self-awareness had the highest correlation index with perceived self-efficacy. Türk (2018) found that high levels of competencies in conflict resolution and empathy correlate directly and significantly with perceived self-efficacy. Similarly, in the present research, we found that conflict resolution and empathy correlate directly and significantly with perceived self-efficacy.

Direct and significant correlations were observed between all evaluated socioemotional competencies and the participants’ level of subjective well-being. The dimensions of motivation and teamwork exhibited the highest correlation indices, followed by the dimension of interpersonal regulation. These findings align with the study conducted by Xu et al. (2020), which found a moderately positive correlation between socio-emotional competencies and subjective well-being. The research by Ogbonnaya (2019) corroborates that experiences of high work demands associated with teamwork practices can have detrimental effects on individuals’ well-being, showing that the relationship between teamwork practices and anxiety is significant and positive.

Antuña (2022) also reported a statistically significant relationship between conflict resolution levels and psychological well-being. These results are consistent with those found in this study, where conflict resolution had the highest correlation index with subjective well-being. Regarding the relationship between empathy and subjective well-being, Wu et al. (2021) found a direct and significant association with an index (*r*) of .48. This aligns with the findings of this research, where the correlation value between the two variables was .30.

In the present study, direct and significant correlations were also found between coping strategies and self-awareness, emotional self-regulation, motivation, and conflict resolution. These results are consistent with those of Piovano et al. (2020), who found that emotional expression was positively correlated with seeking social support, while emotional awareness and prosocial behavior showed negative correlations with avoidance coping strategies and aggressive antisocial actions, respectively. Zyusifa and Affandi (2021) found a correlation index of -.59 between emotional self-regulation and academic stress; these results are consistent with those obtained in the present study. This suggests that as emotional self-regulation competency increases, academic stress levels decrease.

## Conclusion

The purpose of this research was to explore the psychometric properties of the Socio-Emotional Competence Assessment Scale (ECSE) in the Cuban university context, confirming its factorial structure and its utility for measuring socio-emotional competencies. The results reveal a strong relationship between these competencies and key variables such as academic self-efficacy, subjective well-being, and academic stress. It was found that greater development of socio-emotional competencies is positively related to perceived academic self-efficacy, subjective well-being, and adaptive coping strategies for stress. The exclusion of a problematic item improved the quality of the scale, strengthening its validity.

## Limitations

The importance of fostering socio-emotional competencies in university students is highlighted, not only to enhance academic performance, but also to promote emotional well-being and coping abilities. Despite these significant findings, this research has several limitations. First, the use of snowball sampling may affect the representativeness of results. More critically, two essential aspects require future investigation: (a) test-retest reliability analyses to assess the temporal stability of ECSE scores, and (b) predictive validity evidence linking the scale to objective academic performance indicators. These limitations are particularly relevant given the study’s focus on educational applications of the instrument. Future research should address these aspects to strengthen the ECSE’s utility in academic settings.

## Footnotes

* Activities of Meta Platforms Inc. (Facebook and Instagram), and the X Corp. (as the successor of Twitter Inc.), are prohibited in the Russian Federation.

## References

[ref1] Abrahams, L., Pancorbo, G., Primi, R., Santos, D., Kyllonen, P., John, O., & Fruyt, F. (2019). Social-emotional skill assessment in children and adolescents: Advances and challenges in personality, clinical, and educational contexts. Psychological Assessment, 31, 460–473. 10.1037/pas0000591 30869960

[ref2] Anderson, V., Darling, S., Hearps, S., Darby, D., Dooley, J., McDonald, S., …, & Beauchamp, M. (2023). Deep phenotyping of socio-emotional skills in children with typical development, neurodevelopmental disorders, and mental health conditions: Evidence from the PEERS. PLOS ONE, 18. 10.1371/journal.pone.0291929 PMC10566677 37819865

[ref3] Antuña, C. (2022). Bienestar psicológico, inteligencia emocional y resolución de conflictos en miembros de los cuerpos y fuerzas de seguridad del estado español: un estudio correlacional [Psychological well-being, emotional intelligence, and conflict resolution among members of Spanish state security forces: A correlational study]. MLS Psychology Research, 5(2), 123–134. 10.33000/mlspr.v5i2.790

[ref4] Arteaga-Cedeño, W., Carbonero-Martín, M., Martín-Antón, L., & Molinero-González, P. (2022). The sociodemographic-professional profile and emotional intelligence in infant and primary education teachers. International Journal of Environmental Research and Public Health, 19(16), 9882. 10.3390/ijerph19169882 36011515 PMC9407829

[ref5] Barraza-Macías, A. (2018). Inventario sisco sv-21 [SISCO SV-21 Inventory]. Inventario SIStémico COgnoscitivista para el estudio del estrés académico [Systemic Cognitive Inventory for the Study of Academic Stress]. Mexico. http://upd.edu.mx/Piloto/PDF/Libros/Estres.pdf

[ref6] Barrera, U., Schoeps, K., Gil-Gómez, J., & Montoya-Castilla, I. (2019). Predicting adolescent adjustment and well-being: The interplay between socio-emotional and personal factors. International Journal of Environmental Research and Public Health, 16(23), 4650. 10.3390/ijerph16234650 31766641 PMC6926821

[ref7] Baute Abreu, A. C. & Vizcaíno Escobar, A. V. (2023). Autoeficacia percibida en estudiantes universitarios [Perceived self-efficacy in university students]. Tesis para optar el grado de Master en Psicopedagogía [Thesis for the Degree of Master in Psychopedagogy]. https://dspace.uclv.edu.cu/handle/123456789/2964

[ref8] Cavioni, V., Conte, E., Grazzani, I., Ornaghi, V., Cefai, C., Anthony, C., ..., & Pepe, A. (2023). Validation of Italian students’ self-ratings on the SSIS SEL brief scales. Frontiers in Psychology, 14. 10.3389/fpsyg.2023.1229653 PMC10585268 37868591

[ref9] Chen, T. (2022). An investigation and analysis of college English majors’ autonomous learning ability in ubiquitous learning environment. Journal of Environmental and Public Health. 10.1155/2022/9103148 PMC9279108 35844949

[ref10] Cheng, Y. (2020). Academic self-efficacy and assessment. Educational Psychology, 40, 389–391. 10.1080/01443410.2020.1755501

[ref11] Chou Ramírez, L., Vizcaíno Escobar, A. V., Fernández Castillo, E., & López Fernández, R. (2023). Competencias socioemocionales, Aptitud verbal y Creatividad en estudiantes de la Universidad Central “Marta Abreu” de Las Villas [Socioemotional competencies, verbal aptitude, and creativity in students at the Central University “Marta Abreu” of Las Villas]. Tesis para optar el grado de Licenciada en Psicología [Thesis for the Degree of Bachelor in Psychology] (In Press).

[ref12] Çıkrıkçı, Ö., & Odacı, H. (2016). The determinants of life satisfaction among adolescents: The role of metacognitive awareness and self-efficacy. Social Indicators Research, 125, 977–990. 10.1007/S11205-015-0861-5

[ref13] Costa, S., Martínez-Moreno, E., Díaz, V., Hermosilla, D., Amutio, A., Padoan, S., Méndez, D., Etchebehere, G., Torres, A., Telletxea, S., & García-Mazzieri, S. (2020). Belonging and social integration as factors of well-being in Latin America and Latin Europe organizations. Frontiers in Psychology, 11. 10.3389/fpsyg.2020.604412 PMC7756150 33362665

[ref14] Cronin, L., Allen, J., Ellison, P., Marchant, D., Levy, A., & Harwood, C. (2019). Development and initial validation of the life skills ability scale for higher education students. Studies in Higher Education, 46, 1011–1024. 10.1080/03075079.2019.1672641

[ref15] Danner, D., Lechner, C., & Spengler, M. (2021). Editorial: Do we need socio-emotional skills? Frontiers in Psychology, 12. 10.3389/fpsyg.2021.723470 PMC8378804 34421774

[ref16] Dunn, T.J., Baguley, T., & Brunsden, V. (2013). A simulation study on the performance of different reliability estimation methods. Educational and Psychological Measurement, 81(6), 1089–1117. 10.1177/0013164421994184 PMC8451020 34565817

[ref17] Funder, D.C., & Ozer, D.J. (2019). Evaluating effect size in psychological research: Sense and nonsense. Advances in Methods and Practices in Psychological Science, 2(2), 156–168. 10.1177/2515245919847202

[ref18] Gamboa, V., Rodrigues, S., Bértolo, F., Marcelo, B., & Paixão, O. (2023). Socio-emotional skills profiles and their relations with career exploration and perceived parental support among 8th grade students. Frontiers in Psychology, 14. 10.3389/fpsyg.2023.1214395 PMC10445755 37621940

[ref19] Guo, J., Tang, X., Marsh, H., Parker, P., Basarkod, G., Sahdra, B., …, & Salmela-Aro, K. (2021). The roles of social-emotional skills in students’ academic and life success: A multi-informant and multicohort perspective. Journal of personality and social psychology. 10.31234/osf.io/ahg8p 35666915

[ref20] Hu, L.T., & Bentler, P.M. (1999). Cutoff criteria for fit indexes in covariance structure analysis: Conventional criteria versus new alternatives. Structural Equation Modeling, 6(1), 1–55. 10.1080/10705519909540118

[ref21] Huang, C., & Zeng, X. (2023). Social and emotional development of disadvantaged students and its relationship with academic performance: Evidence from China. Frontiers in Psychology, 14. 10.3389/fpsyg.2023.1170656 PMC10203391 37228337

[ref22] Kelley, K. (2018). The MBESS R package (version 4.4.3). http://cran.r-project.org

[ref23] Kim, S., & Shin, S. (2021). Social–emotional competence and academic achievement of nursing students: A canonical correlation analysis. International Journal of Environmental Research and Public Health, 18. 10.3390/ijerph18041752 PMC7916968 33670218

[ref24] Koppenborg, K., Garnefski, N., Kraaij, V., & Ly, V. (2022). Academic stress, mindfulness-related skills and mental health in international university students. Journal of American College Health, 1–9, 787–795. 10.1080/07448481.2022.2057193 35427213

[ref25] López-Barajas, D.M., & Reina-Estévez, A. (2012). Competencias socioemocionales y actitud para la empleabilidad en desempleadas universitarias [Socioemotional competencies and attitude toward employability in unemployed female university graduates]. Revista Española de Orientación y Psicopedagogía [Spanish Journal of Guidance and Psychopedagogy], 23(2), 92–104. http://www.redalyc.org/articulo.oa?id=338230791007

[ref26] Malinauskas, R., & Malinauskiene, V. (2021). Training the social-emotional skills of youth school students in physical education classes. Frontiers in Psychology, 12. 10.3389/fpsyg.2021.741195 PMC8456087 34566822

[ref27] Merlin, I., & Soubramanian, P. (2024). From self-awareness to social savvy: How intrapersonal skills shape interpersonal competence in university students. Frontiers in Psychology, 15. 10.3389/fpsyg.2024.1469746 PMC11404366 39286557

[ref28] Mishra, P., Pandey, C.M., Singh, U., Gupta, A., Sahu, C., & Keshri, A. (2019). Descriptive statistics and normality tests for statistical data. Annals of Cardiac Anaesthesia, 22(1), 67–72. 10.4103/aca.ACA_157_18 30648682 PMC6350423

[ref29] Moradi, A., & Chemelnezhad, M. (2021). Predicting emotional-social competence based on academic engagement, self-efficacy and perception of school climate in high school students. Iranian Evolutionary and Educational Psychology. 10.52547/ieepj.3.4.574

[ref30] Morales-Rodríguez, F., Espigares-López, I., Brown, T., & Pérez-Mármol, J. (2020). The relationship between psychological well-being and psychosocial factors in university students. International Journal of Environmental Research and Public Health, 17. 10.3390/ijerph17134778 PMC7369745 32630816

[ref31] Munir, H., Naz, S., Khan, J., Taj, T., Kashif, M., & Muhammad, D. (2023). Association between socio-emotional competence and self-efficacy of nurse-educators in peshawar. Pakistan Journal of Medical and Health Sciences. 10.53350/pjmhs202317672

[ref32] Nartova-Bochaver, S., Donat, M., Kiral-Ucar, G., Korneev, A., Heidmets, M., Kamble, S. Khachatryan, N., Kryazh, I., Larionow, P., Rodríguez-González, D., Serobyan, A., Zhou, C., & Clayton, S. (2022). The role of environmental identity and individualism/collectivism in predicting climate change denial: Evidence from nine countries. Journal of Environmental Psychology, 84, 101899. 10.1016/j.jenvp.2022.101899

[ref33] Ogbonnaya, C. (2019). Exploring possible trade-offs between organisational performance and employee well-being: The role of teamwork practices. Human Resource Management Journal. 10.1111/1748-8583.12238

[ref34] Palenzuela, D.L. (1983). Construcción y validación de una escala de autoeficacia percibida específica de situaciones académicas [Construction and validation of a perceived self-efficacy scale specific to academic situations]. Análisis y Modificación de conducta [Behavior Analysis and Modification], 9(21), 185–219. https://dialnet.unirioja.es/servlet/articulo?codigo=7101317

[ref35] Piovano, N., Solodovsky, M., & Pascuali, G. (2020). Competencias socioemocionales y estrés. Cómo se relacionan con el rendimiento académico en estudiantes de educación superior [Socioemotional competencies and stress: Their relationship with academic performance in higher education students]. Revista de Investigaciones Científicas de la Universidad de Morón [Journal of Scientific Research, University of Morón], 6. https://repositorio.unimoron.edu.ar/bitstream/10.34073/218/1/5%20

[ref36] Portela-Pino, I., Alvariñas-Villaverde, M., & Pino-Juste, M. (2021). Socio-emotional skills as predictors of performance of students: Differences by gender. Sustainability, 13, 4807. 10.3390/SU13094807

[ref37] Primi, R., Santos, D., John, O., & Fruyt, F. (2021). SENNA inventory for the assessment of social and emotional skills in public school students in Brazil: Measuring both identity and self-efficacy. Frontiers in Psychology, 12. 10.3389/fpsyg.2021.716639 PMC8657760 34899462

[ref38] Repetto Talavera, E. (2009). Formación en competencias socioemocionales: libro del formador [Training in socioemotional competencies: Trainer’s handbook].

[ref39] Rodríguez-Martín, B., & Molerio-Pérez, O. (2012). Validación de Instrumentos Psicológicos Criterios Básicos [Validation of psychological instruments: Basic criteria]. https://www.researchgate.net/publication/328463044

[ref40] Santamaría-Villar, M., Gilar-Corbí, R., Pozo-Rico, T., & Castejón, J. (2021). Teaching socio-emotional competencies among primary school students: Improving conflict resolution and promoting democratic co-existence in schools. Frontiers in Psychology, 12. 10.3389/fpsyg.2021.659348 PMC8249732 34220629

[ref41] Shafait, Z., Khan, M., Sahibzada, U., Dacko-Pikiewicz, Z., & Popp, J. (2021). An assessment of students’ emotional intelligence, learning outcomes, and academic efficacy: A correlational study in higher education. PLoS ONE, 16. 10.1371/journal.pone.0255428 PMC8330937 34343173

[ref42] Shrestha, N. (2021). Factor analysis as a tool for survey analysis. American Journal of Applied Mathematics and Statistics, 9(1), 4–11. 10.12691/ajams-9-1-2

[ref43] Supervía, P., & Robres, A. (2021). Emotional regulation and academic performance in the academic context: The mediating role of self-efficacy in secondary education students. International Journal of Environmental Research and Public Health, 18. 10.3390/ijerph18115715 PMC8198487 34073453

[ref44] Talwar, A., Magliano, J., Higgs, K., Santuzzi, A., Tonks, S., O’Reilly, T., & Sabatini, J. (2022). Early academic success in college: examining the contributions of reading literacy skills, metacognitive reading strategies, and reading motivation. Journal of College Reading and Learning, 53, 58–87. 10.1080/10790195.2022.2137069

[ref45] Türk, F. (2018). An examination of empathic tendency, self-regulation and self-efficacy as predictors of conflict resolution skills in adolescents. Universal Journal of Educational Research, 6, 994–1004. 10.13189/ujer.2018.060520

[ref46] Ullah, M., Akhter, S., Aziz, M., & Islam, M. (2023). Social support: Mediating the emotional intelligence-academic stress link. Frontiers in Psychology, 14. 10.3389/fpsyg.2023.1218636 PMC10509480 37736153

[ref47] Uysal-Bozkir, Ö., Parlevliet, J., & De Rooij, S. (2013). Insufficient cross-cultural adaptations and psychometric properties for many translated health assessment scales: A systematic review. Journal of Clinical Epidemiology, 66, 608–18. 10.1016/j.jclinepi.2012.12.004 23419610

[ref48] Vestad, L., & Tharaldsen, K. (2021). Building social and emotional competencies for coping with academic stress among students in lower secondary school. Scandinavian Journal of Educational Research, 66, 907 – 921. 10.1080/00313831.2021.1939145

[ref49] Vestad, L., Bru, E., Virtanen, T., & Stallard, P. (2021). Associations of social and emotional competencies, academic efficacy beliefs, and emotional distress among students in lower secondary school. Social Psychology of Education, 24, 413–439. 10.1007/s11218-021-09624-z

[ref50] Wang, Y., Yang, Z., Zhang, Y., Wang, F., Liu, T., & Xin, T. (2019). The effect of social-emotional competency on child development in Western China. Frontiers in Psychology, 10. 10.3389/fpsyg.2019.01282 PMC6566918 31231282

[ref51] Wu, W., Qi, Q., Cao, X., Li, S., Guo, Z., Yu, L., …, & Zeng, Y. (2021). Relationship between medical students’ empathy and occupation expectation: Mediating roles of resilience and subjective well-being. Frontiers in Psychology, 12. 10.3389/fpsyg.2021.708342 PMC8503271 34646196

[ref52] Wunsch, K., Fiedler, J., Bachert, P., & Woll, A. (2021). The tridirectional relationship among physical activity, stress, and academic performance in university students: A systematic review and meta-analysis. International Journal of Environmental Research and Public Health, 18. 10.3390/ijerph18020739 PMC7830011 33467118

[ref53] Xia, Y., & Yang, Y. (2019). RMSEA, CFI, and TLI in structural equation modeling with ordered categorical data: The story they tell depends on the estimation methods. Behavior Research Methods, 51, 409–428. 10.3758/s13428-018-1055-2 29869222

[ref54] Xu, X., Pang, W., & Xia, M. (2020). Are emotionally intelligent people happier? A meta-analysis of the relationship between emotional intelligence and subjective well-being using Chinese samples. Asian Journal of Social Psychology. 10.1111/ajsp.12445

[ref55] Yu, M., Brown, T., Hewitt, A., Cousland, R., Licciardi, L., & Lyons, C. (2021). Baccalaureate occupational therapy students’ development of social and emotional competencies. Nurse Education Today, 105, 105032. 10.1016/j.nedt.2021.105032 34198159

[ref56] Zyusifa, R., & Affandi, G. (2021). The relationship between emotion regulation and academic stress in class XII students of vocational high school accounting department. Academia Open, 4. 10.21070/acopen.4.2021.2741

